# Supplementary material for: Are carbon emissions trading and green financial instruments synergistic? -Comprehensive quantitative research based on content analysis
Source: PLoS One. 2024 Mar 7;19(3):e0298601. doi: 10.1371/journal.pone.0298601 (PMC10919601; doi:10.1371/journal.pone.0298601)
Supplement: S1 Data — (ZIP) [file pone.0298601.s001.zip › Data/地级市Z_F工作报告.zip!/║■▒▒/╛ú├┼╩╨/└┤╫╘╣1⁄2╓┌║┼í╛┬φ┐╦╩2╛▌═°í┐_2011.docx]

各位代表：

现在，我代表市人民政府向大会作工作报告，请连同《荆门市经济和社会发展第十二个五年规划纲要(草案)》一并审议，并请市政协各位委员和其他列席人员提出意见。

一、“十一五”时期经济社会发展的回顾

“十一五”时期，是我市经受严峻挑战的五年，也是综合实力和竞争力大幅提升的五年，改革开放取得重大进展的五年，社会事业全面进步和人民群众得到更多实惠的五年。五年来，全市上下紧紧围绕“加快荆门崛起”的总体目标，全力推进新型工业强市、现代农业强市、文化旅游强市和生态宜居城市建设，圆满完成了“十一五”规划目标任务。

（一）综合实力跃上新台阶。2010年全市地区生产总值达到730.07亿元，年均增长13.5%；规模工业增加值330.41亿元，年均增长24.2%；财政总收入79.94亿元，年均增长31.2%，地方一般预算收入23.25亿元，年均增长20.0%；社会消费品零售总额252.98亿元，年均增长19.8%；外贸出口2.98亿美元，年均增长25.5%；累计完成全社会固定资产投资1257亿元、利用外资6.17亿美元，年均分别增长31%、16%；单位生产总值综合能耗、化学需氧量和二氧化硫排放量比“十五”末分别下降20%、7.2%和17.6%；城镇居民人均可支配收入15272元，年均增长12.2%,农民人均纯收入6951元，年均增长13.2%。先后被评为全国科技进步先进市、全国绿化模范城市，被确定为国家循环经济试点市、国家现代林业建设示范市、全国农机化示范区、国家节水型社会试点市、全国创建创业型城市和国家加工贸易产业梯度转移重点承接地。

（二）结构调整迈出新步伐。大力实施工业“3+4计划”，

2010年，石化、磷化、食品、建材、机电、轻纺、新能源等产业可实现销售收入1037亿元，比“十五”末增长2.4倍。规模工业企业达到1173家，增加759家，亿元企业达到188家，增加135家，其中，荆门石化位居全省百亿企业第7位，新洋丰肥业跻身中国民营企业500强。荆门经济开发区、各县市经济开发区、各类特色产业园区及工业重镇建设扎实推进，中粮、中集、中海油、李宁、福耀、雨润、汇源、正邦等“500强”企业和一大批知名品牌相继落户荆门。洋丰肥料、国宝桥米被评为中国名牌和中国驰名商标，广源食品荣膺中国驰名商标。共完成164家市属国有、集体企业改制，省级上市后备企业达到19家。大力实施农业“212计划”，粮食总产连续两年突破50亿斤，市级以上龙头企业达到172家，2010年农产品加工产值达到380.56亿元。农民专业合作社达到2095家，位居全省首位。认证农业“三品”349个。农机综合作业率达到67%。集体林权制度主体改革全面完成。大力实施文化旅游业“133计划”，中心城区运动休闲、京山山水休闲、钟祥文化休闲三大板块基本形成，明显陵等3个景区被评为国家4A级景区，明显陵—漳河景区、大洪山风景区、纪山楚文化旅游区进入鄂西圈十大核心建设景区行列。2010年接待国内外游客865.93万人次，实现旅游总收入40.51亿元。着力搞活城乡市场流通，建成各类市场114个、“万村千乡市场工程”农家店1278家、农村综合服务社813家。金融业健康发展，连续5年获得全省“A级金融信用市”称号。保险、中介咨询等现代服务业发展步伐加快。大力发展循环经济，累计实施循环经济项目380个，完成投资300亿元，初步形成6大循环经济产业链，工业固体废弃物综合利用率达到99%。县域经济发展势头良好，京山包装机械、钟祥农产品加工、东宝磷化等产业被确定为省重点成长型产业集群，京山县、钟祥市连续5年跻身全省县域经济发展20强，东宝区连续2年跻身全省10强，沙洋县2009年度排名前移11位。

（三）城乡面貌发生新变化。大力实施“1311”城镇发展战略，城镇化率由“十五”末的42.3%上升到45.5%。中心城区建成区面积达到50平方公里，人口增加到48万；建成荆门生态运动公园、天鹅湖公园、市体育文化中心等重点项目及象山大道南段、深圳大道西段、凤凰大道、李宁大道等主干道路；中心城区空气环境质量达标率保持在90%以上；连续两次夺得湖北省城镇规划建设管理“楚天杯”和“卫生城市”称号。京山县、钟祥市被评为省级园林城市，沙洋县城市功能不断完善，掇刀区被纳入全省城乡一体化试点。新农村建设扎实推进，孙桥镇、彭墩村、雷都村等一批省、市试点示范镇、村和杨竹流域示范区建设取得阶段性成效，客店镇被评为湖北十大旅游名镇。交通建设快速推进，荆宜、随岳、武荆高速公路荆门段建成通车；完成国省道大中修395公里。积极实施病险水库除险加固、国土整治和大中型灌区节水改造工程，新增和改善灌溉面积165万亩、除涝面积60万亩。电网建设步伐加快，1000千伏荆门特高压变电站、500千伏荆门换流站建成投入运行。夏家湾污水处理厂二期、杨树港污水处理厂、荆门第二生活垃圾卫生填埋场和京山文峰、沙洋、钟祥污水处理厂相继建成投入使用；漳河水库入围国家湿地公园，竹皮河水质逐步好转。完成人工造林134万亩，全市森林覆盖率达到36.9%。

（四）民生建设取得新成绩。坚持每年办好政府10件实事，一批群众关心的民生问题逐步得到解决。累计新增就业18.16万人，扶持8649人创业，城镇登记失业率控制在4.3%以内。五大保险参保总人数达到119.6万人次，基本医疗保险参保对象实现全覆盖。全面启动新型农村社会养老保险试点，落实被征地农民的社会保障待遇3015人，困难企业退休人员医疗保障问题基本解决，惠民医疗制度顺利实施。整村推进式扶贫工作成效显著，累计减少贫困人口近10万人。城乡低保保障标准逐步提高，困难群众临时救助、医疗救助制度全面建立，社会救助体系不断完善。累计筹措资金1.49亿元，资助贫困学生2.77万人次。新建通村公路6273公里，提前一年实现“村村通”目标；累计解决96.42万人的饮水安全问题，完成农村清洁能源入户2万户，改造农村土坯房1.3万户。加强保障住房建设，累计建设廉租住房5966套、经济适用住房8618套，发放廉租住房租赁补贴32789户、3723万元，改造各类棚户区（危旧房）6562户。

（五）社会事业实现新突破。积极推进产学研合作，先后与武汉大学、华中科技大学等高校建立了市校合作关系。着力推进义务教育均衡发展，共落实农村义务教育改革保障经费5.8亿元，九年义务教育完成率达到99.7%。新建、改建、扩建中小学校300余所。组建了荆门职教集团，湖北信息工程学校被确定为全国首批“改革发展示范职高”。荆门职业技术学院与沙洋师范专科学校合并组建荆楚理工学院。大力推进文化体育事业，承办了第八届中国艺术节分会场活动，大型现代湖北花鼓戏《十二月等郎》获“五个一工程”奖、文华大奖和国家舞台艺术十大精品剧目奖；乡镇综合文化站、村级文化活动中心、农家书屋等一批基层文化阵地相继建成，京山县、钟祥市被评为“全国文化先进县”；群众性体育活动蓬勃开展；荆门籍运动员在国际国内重大赛事上共夺得5个世界冠军、9个全国冠军；成功承办湖北省第十三届运动会，并创造了荆门代表团历史最好成绩。积极发展卫生事业，共完成11所县级医疗卫生机构、48个乡(镇)卫生院、1038个村级卫生室标准化建设和改造，基层医疗卫生服务体系进一步完善；全力应对手足口病、“三鹿”奶粉事件、甲型H1N1流感等突发公共卫生事件，艾滋病、结核病、血吸虫病等重大传染病和地方病的防治得到加强。着力推进医药卫生体制改革，国家基本药物制度试点稳步实施，公立医院改革试点工作有序进行。深入开展高危行业、食品药品、安全隐患排查和专项整治工作，安全生产形势持续稳定好转。低生育水平进一步稳定，年均人口自然增长率为3.98％。。耕地保护得到加强。精神文明创建活动深入开展，市民文明素质明显提升。中心城区社区建设“三基”行动计划成效显著。深入开展国防教育，双拥共建工作卓有成效，民兵基层建设水平明显提升，人民防空工作稳步推进。率先完成南水北调中线工程移民外迁安置任务。抗震救灾对口支援任务圆满完成，援疆工作成效明显。统计、物价、工商、烟草、外事侨务、台湾事务、仲裁、气象、防震、地方志、档案、知识产权、地质灾害防治、应急管理、住房公积金管理等工作不断发展，民族宗教、广播电影电视、新闻出版、工会、青年、妇女、儿童、老龄、慈善、残疾人等事业取得较大进步。

（六）民主法制建设取得新成效。深入开展“五五”普法活动。坚持依法行政，自觉接受市人大、市政协监督，累计办理人大议案建议515件、政协提案1015件。认真开展深入学习实践科学发展观和“创先争优”活动，扎实推进“能力建设年”、“作风建设年”活动，政府自身建设进一步加强。政府机构改革基本完成，事业单位改革稳步推进。继续深化行政审批制度改革，加强电子政务建设，积极推进政府信息公开，行政效能进一步提高。积极开展文明执法教育活动，行政执法行为进一步规范。以规范招投标监管和深入开展“小金库”、工程建设领域突出问题专项治理为契机，推行重大项目跟踪审计，强化监察审计监督和制度建设，大力开展反腐倡廉工作，廉政建设和反腐败工作取得新成效。认真处理群众来信来访，我市化解信访积案工作经验在全国推广。“平安荆门”建设成效显著，沙洋县被评为全国平安建设先进县。“两实全覆盖”有效推进，群防群治组织建设和工作机制不断完善，预防和打击违法犯罪活动整体效能不断提升，全市社会和谐稳定、人民安居乐业。

刚刚过去的2010年，是我市发展史上不同寻常的一年。面对经济形势复杂多变、区域竞争更加激烈、调整转型矛盾突出等严峻考验，全市按照“千亿目标、百亿增长”的要求，坚定不移地调结构、稳增长，一手抓总量扩张、一手抓转变发展方式，经济社会保持了平稳较快发展的良好态势；坚定不移地办省运、促发展，统筹推进经济建设、城市基础设施建设和全民体育活动的开展，向全省人民奉献了一届高水平、有特色的省运盛会；坚定不移地抓规划、谋长远，以编制“十二五”规划为契机，谋划了一批支撑长远发展的重大项目；坚定不移地惠民生、保稳定，以办好政府10件实事为抓手，在共建共享中促进社会和谐。2010年，全市地区生产总值增长15.6%，规模工业增加值增长25%，全社会固定资产投资增长41.4%，社会消费品零售总额增长20.8%，地方财政总收入增长32.5%，地方一般预算收入增长28.2%，城镇居民人均可支配收入、农民人均纯收入分别增长10.2%和16.7%，全面完成了年初确定的各项目标任务。

各位代表，五年来，面对国际国内环境的巨大变化、金融危机的巨大冲击和自然灾害频发的不利影响，我们之所以能够战胜一个又一个困难，保证荆门改革开放和现代化建设的航船破浪前进，靠的是科学发展观指导下的创新实践，靠的是省委、省政府和中共荆门市委的正确领导，靠的是市人大、市政协的监督和支持，靠的是全市人民的团结奋斗、顽强拼搏。在此，我代表荆门市人民政府，向全市干部群众，向各民主党派、工商联、无党派人士和人民团体，向人大代表、政协委员，向驻荆人民解放军、武警官兵和公安干警，向驻荆中省单位，向关心、支持荆门发展的社会各界朋友，致以崇高的敬意和诚挚的感谢！

回顾五年工作，我们深切体会到，要把荆门各项事业顺利推向前进，必须始终把解放思想作为开创新局面的“总开关”，切实落实到发展目标的确定上、落实到具体政策的制定上、落实到工作的操作层面上；必须始终把科学发展作为首要任务，以循环经济为抓手，以新型工业化为突破口，优化产业结构，加速转变经济发展方式；必须始终把改革开放作为加快发展的强大动力，以深化改革突破体制障碍，以扩大开放增创发展优势；必须始终把改善民生作为一切工作的出发点和落脚点，使改革发展的成果更多地惠及于民；必须始终把抓落实、求突破、见成效作为提升政府执行力的立足点，创造对得起人民、无愧于历史、经得起检验的政绩。

在充分肯定成绩的同时，我们也清醒地看到，我市经济社会生活中还存在不少问题和挑战，主要表现在：经济结构还不够优化，主导产业带动能力不够强，传统产业比重偏高，农业基础设施薄弱，要素制约和资源环境约束压力较大；城镇化水平不够高，城市规划建设管理滞后，中心城区个人违规建房查处不力；物价上涨压力加大，就业、医疗、住房、交通、环境等涉及群众切身利益的问题有待进一步解决，社会矛盾相互交织，维护和谐稳定的任务更加繁重；政府职能转变还不够到位，行政效能和公务员素质有待提高，廉政建设仍需加强，等等。对于这些问题，我们将采取更加有效的措施，认真加以解决。

二、“十二五”时期的指导思想和目标任务

“十二五”时期的五年，是我市实现跨越发展、加快崛起、全面建成小康社会的关键期，是深化改革开放、加快转型升级的攻坚期，是统筹城乡发展、推进城乡一体化的加速期，市政府根据《中共荆门市委关于制定经济和社会发展第十二个五年规划的建议》，制定了《荆门市经济和社会发展第十二个五年规划纲要（草案）》，提出了“十二五”时期全市经济社会发展的指导思想和发展目标。

指导思想是：高举中国特色社会主义伟大旗帜，以邓小平理论和“三个代表”重要思想为指导，全面贯彻落实科学发展观，紧紧围绕全面建成小康社会、实现荆门跨越崛起的奋斗目标，坚持以科学发展为主题，以加快转变经济发展方式为主线，以经济结构调整为重点，以民生改善为根本出发点，以解放思想、改革开放为动力，深入推进新型工业强市、现代农业强市、文化旅游强市和生态宜居城市建设，突出抓好产业转型升级、循环经济发展和新型城镇化建设三大战略任务，努力把荆门打造成为全国新型化工基地和优质农产品生产加工基地，中部地区先进装备制造业基地和生产性现代物流基地，区域性文化旅游休闲度假区，加快建成全国有重要影响的循环经济示范市。

主要目标是：全力完成“三大计划”，努力实现“五大目标”。

“三大计划”，即经济总量“倍增计划”，主要经济指标在2010年的基础上实现翻番；单位能耗、排放和农村人口“减量计划”，节能减排任务圆满完成，农村居住人口大幅减少；社会保障“覆盖计划”，养老、医疗、保障住房和最低生活保障实现城乡全覆盖。“五大目标”，即经济实力显著增强，经济结构显著优化，生态环境显著改善，改革开放步伐显著加快，人民生活水平显著提升。

“十二五”时期，全市主要经济指标增幅力争高于全省平均水平、高于“十一五”发展水平，综合经济实力在全省位次前移。到2015年，全市三次产业结构比重调整为11∶54∶35，生产总值年均增长15%，规模以上工业增加值年均增长18%，固定资产投资年均增长20%以上，地方一般预算收入年均增长18%，城镇居民人均可支配收入年均增长12%，农民人均纯收入年均增长13%，单位生产总值综合能耗有效降低，主要污染物排放指标达到省定要求，城镇化率达到60%，累计新增城镇就业20万人。

确定上述指导思想和主要目标，充分体现了推进荆门科学发展的要求，充分体现了省委、省政府对荆门加快发展的希望，充分体现了市委关于经济社会发展的总体思路和全市人民的共同愿望，充分体现了荆门跨越发展、加快崛起的客观需要和现实可能。《纲要（草案）》及《说明》已印发给各位代表，请大家审议。在具体实施中，我们将牢牢把握以下几项原则：

——坚持加快发展，壮大经济实力。始终把“好”与“快”有机统一起来，积极争取和实施一批大交通、大物流、大产业、大生态建设项目，促进投资较快增长。认真落实国家扩大内需战略，不断扩大消费需求。积极转变外贸发展方式，进一步挖掘出口增长潜力。切实加强各类园区建设,使之成为项目建设的平台、对外开放的窗口、产业升级的高地和城镇发展的新区。大力实施“市场主体增量计划”，深入推进全民创业工程，培育发展一批年销售收入过50亿元的大企业、大集团。科学规划中心城区产业定位和空间布局，强化资源整合，推动中心城区经济社会快速发展，形成具有较强竞争优势的二、三产业集聚区。以工业化、城镇化、农业现代化为方向，加快形成各具特色的县域经济。

——坚持转型发展，优化经济结构。把经济结构调整作为加快转变经济发展方式的主攻方向，改造提升传统产业，积极发展石化、磷化、食品、建材、机电、轻纺及体育用品等产业，全力打造千亿级化工和千亿级农产品加工业。培育发展高新技术和战略性新兴产业，突破性发展通用航空和新能源产业，大力发展节能环保、新材料、生物医药、电子信息等产业，加快形成先导性、支柱性产业。以农业产业化、农业机械化、农村城镇化、农村服务社会化为重点，大力发展现代农业，努力形成具有荆门特色的现代产业体系和发展模式。深入推动文化旅游业和现代物流业发展，积极发展电子商务等高端服务业，改造提升商贸、餐饮等传统服务业，力争服务业增加值比重和从业人员比重显著提高。

——坚持创新发展，增强内生动力。扎实推进“创新荆门”建设，优先发展教育事业，积极实施人才强市战略，增强科教和人才对经济的支撑力。立足鄂西圈、融入武汉圈，加强区域合作，提高利用外资能力和水平，着力建成中部重要的国内外产业、资本转移承接地。以更大的决心和勇气推进重点领域和关键环节的改革，进一步创新投融资、土地集约利用、环境保护等机制，创新政府管理，提高行政效能。着眼于破解城乡二元结构，大力拓展中心城区空间，完善城市功能，扎实推进县城扩容提质,积极开展镇级市试点，统筹推进新农村建设,促进城乡一体化发展。全面加快公路、铁路、航空、水运、电力等基础设施建设，努力把荆门建成鄂中区域性综合交通枢纽。

——坚持循环发展，建设生态文明。大力推广低碳技术，切实抓好循环产业园区建设，延伸循环经济产业链条，推动社会循环经济发展，倾力打造荆门循环经济“金字招牌”。推进重点领域和企业的节能减排工作，严格控制污染物排放总量。加强资源节约和管理，狠抓生态环境保护和治理，大力发展现代林业，积极创建国家卫生城市和环保模范城市，努力建设山清水秀天蓝的生态宜居城市。

——坚持和谐发展，保障和改善民生。努力扩大就业再就业，不断提高城乡居民收入，完善社会保障体系；加快医疗卫生等社会事业发展，不断繁荣发展文化事业和文化产业；加强和创新社会管理，促进公平正义，切实做到学有所教，积极保障病有所医，加快推进老有所养，着力促进住有所居，让人民群众共建共享改革发展成果。

三、扎实做好2011年的各项工作，努力实现“十二五”良好开局

2011年是实施“十二五”规划的第一年，是中国共产党建党90周年，也是本届政府任期的最后一年。做好今年的工作，对于“十二五”起好步和七届政府收好官，至关重要。今年经济社会发展的主要预期目标是：全市地区生产总值增长13%；规模以上工业增加值增长22%；全社会固定资产投资增长25%；居民消费价格涨幅控制在4%左右；社会消费品零售额增长20%；地方一般预算收入增长18%；出口总额增长18%，外商直接投资增长12%；城镇居民人均可支配收入增长10%；农民人均纯收入增长10%；城镇登记失业率控制在4.3%以内；人口自然增长率控制在4.5％。以内；单位生产总值综合能耗、主要污染物排放量削减率完成省定目标。

做好今年的各项工作，必须按照市委六届十一次全会精神，紧紧围绕荆门跨越崛起的目标，从以下六个方面狠下功夫，奋力加快发展，着力改善民生，努力维护稳定，确保实现“十二五”良好开局。

（一）全力推进投资、消费和出口增长，增强经济发展动力

继续加大投资力度。始终坚持把重大项目建设作为扩大投资的首要载体，坚定不移地大抓项目、抓大项目。全年开工建设206个亿元项目，年内完成投资290亿元，其中重点调度40个重大项目，完成年度投资94亿元。确保航特铸造二期、格林美电子废弃物循环利用工程、雪驰服装加工一期、众和纺织三期、京山潭龙石材二三期、钟祥广源食品工业园等项目建成投产，加快推进中石化荆门分公司扩能改造、深加工及成品油管道工程、李宁加工园及材料园、华能热电联产、京山伟嘉纺织工业园、沙洋正邦养殖加工、大峪口磷复肥、德力西电气设备制造等项目建设，做好荆门电厂四期扩建、非织造材料产业园（布谷）、沙洋纺织孵化园、钟祥汉江二桥、碾盘山水利水电枢纽工程等40个项目的前期推进工作。抓好中央投资项目争取和项目储备，形成能够有效引导我市“十二五”项目建设的项目库。

努力扩大消费需求。认真落实国家关于收入分配和促进消费的各项政策，着力推进“家电下乡”及“家电以旧换新”，加快“新农村现代流通服务网络工程”建设，引导支持承办企业加快配送中心建设和农家店信息化改造。抓好“双百市场工程”，积极推动“农超对接”，加快南台、惠民、蔡湾等城区菜市场标准化改造。进一步推进教育、文化、娱乐、健身、家政消费，引导汽车等大宗商品消费，大力拓展旅游消费。加快掇刀大型现代综合农贸市场、屈家岭农产品交易市场等项目建设，促进中天街商业集聚区做大做强，活跃城乡物资交流。

大力发展开放型经济。着力优化投资环境，认真策划招商项目，加大招商引资力度，力争外商直接投资1.64亿美元，引进市外境内资金突破200亿元。积极承办中国光彩事业荆门行活动，进一步加强与国内外客商的合作。创造本土企业与引资企业一视同仁、公平竞争的政策环境，促进民营经济快速发展。引导各类企业积极开拓国际市场，壮大出口队伍，扩大出口规模，力争年出口1000-3000万美元的企业达到5家，3000万美元以上的达到3家。引导具备条件的企业大胆“走出去”，到境外设立销售网络或投资办厂。以钟祥新世纪外经公司为龙头，培育全省重点外派劳务基地品牌。

扎实做好财税金融工作。加强财源建设，优化财源结构，加快做大财政“蛋糕”。坚持依法治税，不断挖掘税收潜力，努力提高税收收入占财政收入的比重。牢固树立过“紧日子”的思想，坚决制止铺张浪费，严格控制公用开支，确保把有限的资金用于加快发展和改善民生。完善金融组织体系，全市4家农村信用联社改制为农村商业银行，引进3家左右股份制商业银行，组建5家村镇银行、3—5家小额贷款公司。大力发展担保业，推进政策性保险试点。政府出资引导，吸引社会资本组建创投企业。强化金融生态环境建设，突出完善农村支付服务环境，确保信贷投放增速高于全省平均水平和全市生产总值增长水平，中小企业贷款增速高于全市贷款增速。支持凯龙、新洋丰等企业加快上市步伐。促进保险业健康发展。

（二）全力推进结构调整，加速主导产业优化升级

加快推进新型工业化。紧紧围绕发展两个千亿级产业目标，着力扩规模、调结构，提升产业竞争力。出台支持工业企业技术改造和科技企业“孵化器”建设的政策措施，引导企业加大技改投入，积极申报国家级、省级重点项目，实施重点技改项目100个以上。以发展产业链经济为重点，加大产业整合力度，促进主导产业集群发展，力争石化、磷化、食品、建材、机电、轻纺、新能源、航空等产业完成产值1300亿元，其中化工和农产品加工业分别达到650亿元、430亿元。加快经济开发区、各类特色园区及工业重镇建设，推动荆门经济开发区进一步拓展发展空间，完善运行体制和机制，搭建投融资平台，狠抓招商引资和项目建设，做大做强李宁产业园、格林美循环产业园和中集、福耀现代装备制造等专业园区，培育税收过千万元的企业6家以上，努力创建国家级开发区。积极发展战略性新兴产业，加快特种飞行器研发制造园、通用航空产业园等项目前期推进工作，抓好华山观风能电场、屈家岭安能生物质发电项目建设，支持东宝森工科技产业园不断延伸产业链条、加快发展。启动“50强企业成长路线图计划”，支持优势企业在兼并重组、裂变扩张上迈出新步伐，力争销售收入过10亿元的企业达到12家，其中50亿元以上企业2家。实施中小企业成长工程，加快“小进规”步伐。选择30家科技型中小企业进行重点培育，力争新增高新技术企业5家，高新技术产业增加值占规模工业增加值的比重达到14%以上。强化煤电油气运等生产要素的保障，促进企业达产达效。

大力发展现代农业。调整种植业结构和区域布局，加快国家大型商品粮基地项目建设，巩固优质稻生产基地200万亩、“双低”油菜基地200万亩、优质棉基地50万亩。切实抓好“菜篮子”工程建设，启动10个核心示范基地和汉江百里蔬菜产业走廊建设，力争新增蔬菜基地面积3.5万亩，达到46万亩，确保市场供应充足，菜价基本稳定。大力发展畜牧水产业，创建省级以上畜禽养殖标准化示范场30个，发展高效渔业养殖模式85万亩。继续推进京山桥米产业、沙洋食品工业、钟祥农产品加工等园区建设，力争农产品加工产值与农业总产值之比达到1.6∶1，新增国家级龙头企业1家，省级龙头企业8家，全年新认证农业“三品”30个。加快推行标准化生产，进一步健全农业技术推广和动植物疫病防控体系，从生产、流通、销售等环节加大农产品质量安全检测、监管力度。深入推进农业机械化，提高农机配套水平和使用效率，力争农机综合作业率达到68%。大力发展农民专业合作社，进一步扩大规模，增强服务带动功能。加强农业基础设施建设，完成投资5亿元，建设重点水利工程项目160个，改造中低产田5万亩。引导和鼓励发展各类农产品流通合作组织、协会，发展运销大户和农民经纪人队伍。深化集体林权制度改革和国有农场改革，巩固和完善农村公益性服务“以钱养事”机制。依法、有序推进土地流转，积极发展多种形式的适度规模经营。认真落实中央、省、市各项惠农政策，增强农业发展动力。

积极发展现代服务业。着力打造“三大旅游板块”，加快推进明显陵─漳河景区、大洪山等核心景区和京山绿林世纪广场、沙洋楚国城、钟祥明文化主题公园、东宝仙居乡村游、掇刀喜民园、屈家岭月湖度假村等重点旅游项目建设；支持京山争创全省、全国旅游强县，绿林、漳河争创全省旅游名镇；启动绿林山和明显陵景区创5A级、象山风景区创4A级景区工作；加大旅游营销力度，办好油菜花节、长寿文化节等特色旅游节庆活动，力争全年接待国内外游客1000万人次，旅游总收入突破50亿元。大力发展现代物流业，建设京山、沙洋、钟祥三个综合物流园区，支持掇刀创建全省首批现代服务业示范区，加快推进星球商业、九禾、东方海外、上海商贸城等物流项目，改造提升洋丰、楚元石化等企业自营物流，促进各类物流企业做大做强。规范和完善土地供应，调整住房供应结构，保持房地产投资的合理增长。围绕服务主导产业和开发区发展，重点发展金融、中介、检验检测、信息等生产性服务业。

（三）全力推进循环经济，着力保护生态环境

大力发展循环经济。加快荆门化工、格林美电子废弃物、荆襄磷化等循环经济园区建设，不断完善主干道、水、电等配套基础设施。加快荆门石化3万吨/年硫磺回收装置、新洋丰肥业3×20万吨/年硫磺制酸余热回收利用、天茂20万吨/年碳四制丙烯工程等项目建设，力争全年实施循环经济项目100个，完成投资100亿元。围绕石化、磷化、建材、轻纺、农产品加工和新能源等产业的延伸配套，着力构建具有荆门特色的循环经济产业体系。加快发展生态循环农业，积极推广秸秆养牛—牛粪种菇—菇料还田、虾稻连作、猪—沼—稻—渔等高效生态农业生产模式，推广农业循环经济模式10万亩。加快推进农村能源入户工程，力争新增2万户。完善再生资源回收利用体系，推进资源再生利用产业化。

加强资源节约和管理。大力实施国家“十大重点节能工程”，组织实施节能工程50个以上。抓好磷矿、煤矿、石膏等资源的合理保护和有效利用，推进共伴生矿产、尾矿及磷石膏、煤矸石等固体废弃物的综合利用。加大城区周边石膏采空区治理力度。大力开展土地卫星图片执法检查工作，认真落实耕地保护和土地管理制度，实施汉江流域土地整治25万亩以上。推进节水型社会试点，强化水资源管理和有偿使用，鼓励企业循环用水，实现零排放。做好全国水利普查工作。

加强生态环境保护和治理。继续实施主要污染物减排目标责任制管理，严格环境准入制度，完成省下达的淘汰落后产能任务。采取沿河乡镇建污水处理厂、汉江入口建湿地、综合治理支流和增加生态补水量等措施，加大竹皮河综合治理力度。编制漳河水源地保护规划，用最严格的措施保护漳河。加强长湖环境保护，着手治理长湖污染。加强城镇集中饮用水源保护。综合治理中心城区粉尘、废气、噪声、汽车尾气污染，启动粉尘重点污染源改造搬迁工作。全面推行企业清洁生产和排污许可制度，加强危化企业和辐射环境管理。高度重视农村环境保护工作，严禁水库投肥养鱼，大力防治畜禽集中养殖污染，有效降低农村面源污染。积极推动现代林业建设，全面开展省级、国家级森林城市创建活动，力争全市新增造林面积20万亩以上。大力倡导低碳生活和绿色消费，在全市推广高效绿色照明产品7万只。

（四）全力推进新型城镇化建设，加快城乡一体化进程

大力实施“1331”城镇发展战略，加快形成以中心城区为龙头、3个县城为支撑、3类重镇（镇级市、工业重镇、旅游三产重镇）为结点、1000个左右新型农村社区为基础的城镇化和城乡一体化新格局。

完善城乡规划体系。大力推进“数字荆门”建设，建立荆门市地理信息中心。启动新一轮荆门市城市总体规划修编，加大中心城区控制性详规编制力度。高起点、高标准、大手笔做好西城区新区规划,重点做好中央商务区、荆门火车站客运中心片区和新市级行政服务功能区等重点区域的城市设计；着力做好高速公路出入口、重要道路交叉口等城市节点的景观改造规划。进一步完善高新技术产业园、化工循环产业园、航空产业园等重要园区的规划。加强城镇、村庄规划工作，实现建制镇总体规划编制全覆盖，村庄建设规划编制覆盖率达80%。严格规划监管，保障规划实施，对中心城区规划区范围内实行规划集中统一管理。

强化中心城区建设和管理。启动西城区建设，新建“两横（漳河大道、双喜大道）三纵（长坂坡路、天鹅路西段、西外环）”干道，拉开西城区道路骨架。加快建设南城区，拉通龙井大道、掇刀石大道等主干道，建成李宁大道北段、物流园路、李宁六路、花港路等道路，完善南城区路网结构。改造提升老城区，完成5个主要交叉路口综合改造，开展竹皮河城区段后续整治，完善老城区主干道刷黑工作。加快编制城中村改造规划，制定配套政策，启动综合改造工作。合理划分市、区两级事权和责权，加强交通秩序、市容环境和户外广告等专项治理。坚决整治违法违规建设，强力推进拆违控违工作。大手笔实施城区绿化美化，高品位绿化漳河大道，改造完善象山风景区，建成开放东宝山—千佛洞风景区，新建凤凰公园、双严河公园和银河游园，积极创建国家园林城市。

推进城乡基础设施一体化建设。着力完善路网结构，加快推进荆门至沙洋一级公路、京山三阳至屈家岭旅游大环线建设，启动武荆高速至京山城区一级公路和屈家岭连接线建设，完成国省道大中修100公里，县乡道改造100公里，新建五级客运站5个，高水准规划实施武荆高速荆门段沿线绿化工程。大力推进轨道交通建设，支持荆沙铁路改造项目，启动荆门火车站客运中心建设，做好武汉城市圈城际铁路延伸至京山的项目前期工作。积极发展水运事业，启动石牌核心港区及配套物流园、工业园规划工作，支持沙洋中心港区尽快对接武汉—沙洋—荆州千吨级航道圈。

发展壮大县域经济。认真落实促进县域经济发展的各项政策措施，支持各县（市、区）大力发展产业集群和特色产业，进一步壮大经济实力，努力实现保强进位目标。支持各县（市）城区加快中等城市建设步伐，抓好东宝区统筹城乡和屈家岭现代农业示范区建设，支持掇刀区争当全省城乡一体化发展的排头兵。加强中心城区卫星镇建设，支持十强工业重镇和十大旅游三产重镇加快发展。引导人口向城镇转移、产业向园区集中，以人口和产业集聚带动县城规模扩大和中心城镇发展。

加强新农村建设。切实抓好杨竹流域生态文明新农村及省、市新农村试点示范镇、村建设。以中心村为重点，以新型农村社区为抓手，按照“宜居村庄”的标准和要求，在全市建设26个农村新社区或中心村。加强乡村公路、安全饮水工程等基础设施建设，加快实施新一轮农村电网改造升级工程，增强农村环境综合整治实效，进一步改善农民生产生活条件。发展壮大村级集体经济，不断增强新农村建设内生动力。

（五）全力推进各项社会事业发展，构建和谐荆门

努力促进就业。认真落实全民创业政策，通过开发岗位、强化培训、增加投入、加强服务等措施，扎实推进国家级创业型城市建设。继续实施“三支一扶”计划，完善“市毕业生就业促进联盟”运行机制，促进充分就业。实施就业援助政策，深入开展城镇“充分就业社区建设”，加快实施农村劳动力转移工程和技能就业计划，抓好新生代农民工的培训和就业工作，帮助下岗失业人员再就业1万人，农村劳动力转移就业5万人。

不断提高社会保障水平。加大社保扩面征缴力度，力争参保人数新增2.65万人次。逐步扩大新型农村养老保险范围。巩固发展新型农村合作医疗制度，在全市范围内实现门诊统筹全覆盖。完善社会救助体系建设，建立物价上涨与低收入群众生活补贴联动机制，推进城乡低保动态管理、应保尽保和困难群众临时救助、医疗救助全覆盖。启动“千福园”老年公寓二期、市残疾人托养康复中心工程建设。建立依托“12343”社区服务和“96580”家政服务热线，完善社区养老和居家养老、家政服务信息网络。扎实推进保障住房建设。切实做好防通胀、稳物价工作，整顿和规范市场价格秩序，努力保持物价基本稳定。

大力发展科教、文体、卫生事业。进一步深化产学研合作，加快风险投资引进工作，支持有条件的企业创建国家级、省级技术中心、研发中心、工程中心或重点实验室。大力加强各类人才队伍建设，培养和引进一批现代产业领军人才和高素质技能型人才。积极创建全国科普示范县（市）。制订和实施荆门中长期教育改革发展规划纲要，推动教育科学发展。启动义务教育阶段学校标准化建设，继续实施中小学校校舍安全工程，完成所有D级危房改造任务。依托荆门职教集团，启动职教产业园建设。继续支持荆楚理工学院建设发展。整体规划全市学前教育，提高幼儿教育教学水平。继续实施文化惠民工程，大力发展文化产业，支持荆门日报传媒集团做大做强，加快组建广播影视传媒集团和荆门文化体育产业集团，启动荆门市工人文化宫重建工程。依托荆门生态运动公园和体育文化中心，积极创建国家级全民健身基地，全力做好绿林山国家登山训练基地争取工作，大力推进全民健身运动和竞技体育发展，支持京山县创建全国网球之乡。继续深化医药卫生体制改革，认真做好市二医公立医院改革试点工作，健全基层医疗卫生服务体系。高标准规划建设南城区综合三甲医院，改造石化医院。为1000名贫困白内障患者免费进行复明手术，对全市134例0-6岁贫困残疾儿童实施抢救性康复工程。

强化社会管理创新。加强政府应急指挥平台和救援能力建设，推进人防和防震减灾工作，努力提高应急管理水平。加强和改进社区工作，推进“三基”建设向县（市）城区和城镇延伸，进一步提高社区工作和服务水平。支持掇刀区加快全省社会管理创新试点建设。组织好第八次村委会和第四次居委会换届选举工作。注重互联网等新兴媒体的建设、应用和管理，积极回应和引导网络民意。进一步健全重大决策、重大项目社会稳定风险评估机制，避免引发新的矛盾。健全社会矛盾纠纷排查调处机制，进一步规范信访秩序，引导群众理性合法表达利益诉求，着力构建大调解、大信访工作格局。严格落实安全生产责任制，强化煤炭、化工、建筑以及特种设备等重点行业的安全监管，扎实开展全省食品药品安全先进市创建活动，切实做好校园安全和周边环境专项治理工作。大力推进社会治安综合治理，建立健全社会治安动态防控体系，着力整治社会治安突出问题；继续深入开展复式执法活动，有效化解社会矛盾，积极预防、坚决打击违法犯罪，切实增强人民群众的安全感。

协调发展各项事业。做好人口与计划生育工作，稳定低生育水平，提高人口素质。全力推进扶贫开发，积极扶持老区加快发展。大力支持驻荆部队建设，抓好国防动员和民兵预备役工作，认真落实军人及军属优抚安置政策，做好军队转业干部、复员退伍军人接收安置工作。深入开展双拥共建活动，增强军政军民团结。全面落实移民后期扶持政策，做好南水北调工程移民安置各项后续工作。继续开展对口援疆工作。进一步完善住房公积金制度。深入抓好市第二届地方志编辑出版工作。关心支持工会、共青团、工商联、妇女、儿童工作，积极推进民族宗教、广播电影电视、新闻出版、外宣、老龄、慈善、残疾人、档案、外事侨务、台湾事务、通信、邮政、烟草、盐业、仲裁、气象等社会事业发展。

各位代表，今年我们将继续为群众办好10件实事：

1.启动三水厂管道取水工程建设，提高中心城区安全供水保障能力。

2.全年实现城镇新增就业4.3万人，通过政策扶持创业5000人，带动就业2万人。

3.新增或改扩建乡镇中心幼儿园30座；资助100名应届荆门籍贫困大学新生入学；对中等职业学校8800名城乡家庭经济困难学生进行资助；对市特殊教育学校所有在校学生实行免学杂费、免课本费、免住宿费和免生活费的“四免”政策。为困难群众及特殊群体免费提供法律援助。

4.加大住房保障力度，新增廉租住房1220套，新增廉租住房租赁补贴1400户；建设经济适用住房1415套；建设公共租赁住房700套；改造棚户区20万平方米。加强中心城区老年公寓建设，新增床位500张。

5.硬化、改造田间渠系500公里；解决农村10万人安全饮水问题。

6.新建通村公路200公里，通组公路路基2000公里，碎石路面1000公里；改造升级县乡道100公里；新建候车棚100个，招呼站100个。

7.发展村级综合服务社400家；为农民提供丰产仓1万套。

8.建设村级文化活动中心60个、农家书屋200个、农民体育健身工程200个；组织开展100场文化惠民文艺演出活动。

9.新增或调整公交线路5条，新投放公交车60辆以上；新改造20条小街小巷，完成杨湾桥重建和东宝山隧道维修工程；对部分路段设施老化的路灯进行改造，新建10条小街小巷路灯。

10.实现城镇职工和居民医疗保险市级统筹；年底前，全市所有政府举办的基层医疗卫生机构实施基本药物制度，实现零差率销售。

（六）全力推进政府自身建设，建设服务政府和法治政府

更加注重提高政府执行力。坚持市委统一领导，认真执行市人大及其常委会的各项决议，依法自觉接受监督；主动听取人民政协的意见与建议，积极支持政协履行职能。切实做好人大议案、代表建议和政协提案办理工作，做到件件有着落、条条有办复。建立健全政府工作落实机制，完善部门之间协调配合机制，实行更加严格的督查督办和责任追究制度，使每项工作任务到人、落实到位，切实做到事有专管之人、人有明确之责、责有限定之期，确保政令畅通。

更加注重提高政府创新力。以改革创新精神推进政府自身建设，确保政府一切工作服从服务于发展的需要、服从服务于公平与效率的需要、服从服务于改善民生的需要。大力开展“解放思想、跨越发展”主题实践活动，切实把解放思想落实到解决具体问题上来，全力打造荆门“精神区位”优势。继续开展能力建设年活动，大力提倡勤于学习、善于调研、勇于创新的精神，强化“能力席位”意识，着力提高领导干部谋划发展、统筹协调、公共服务、社会管理、维护稳定的本领，着力提高公务员的操作能力。深化行政审批制度改革，进一步削减行政审批事项，健全和完善首席代表制、并联审批制度，提高审批效率，为各类经济主体创造机会均等、公平竞争的市场环境和社会环境。

更加注重提高政府公信力。坚持依法行政，规范执法行为，促进严格、公正、文明执法。建立涉及群众切身利益的重大决策群众参与制度、重大城建和招商项目星期六例会制度，切实完善公众参与、专家论证和政府决策相结合的决策机制。政府出台的政策、作出的承诺必须兑现，做到言必信、行必果。完善政府信息公开机制，积极推进电子政务，打造“阳光政府”。不断加强作风建设，坚决反对形式主义、官僚主义，以良好作风影响和带动群众。加强反腐倡廉建设，认真贯彻《中国共产党党员领导干部廉洁从政若干准则》，筑牢公务员队伍拒腐防变的思想道德防线，促进廉洁从政。严格落实“一岗双责”和党风廉政建设责任制，着力建设完善惩治和预防腐败体系。严格执行“收支两条线”管理规定，切实加强对各种行政性收费及罚没收入的监管。强化对行政权力运行的制约和监督，健全完善政府投资财政评审和审计监督、领导干部经济责任审计等制度，加强对财政资金运行、重大项目建设等重点环节和部位的有效监督，建立公共资源交易中心，加强对招投标市场的综合监管，建设廉洁政府。

各位代表！新的起点孕育新的希望，新的希望承载新的使命。让我们紧密团结在以胡锦涛同志为总书记的党中央周围，在中共荆门市委的坚强领导下，紧紧依靠全市人民，高举中国特色社会主义伟大旗帜，深入贯彻落实科学发展观，解放思想、锐意创新，抢抓机遇、扎实工作，为实现荆门跨越发展、加快崛起而努力奋斗！
